# Supplementary material for: Modeling glioblastoma heterogeneity as a dynamic network of cell states
Source: Mol Syst Biol. 2021 Sep 16;17(9):e10105. doi: 10.15252/msb.202010105 (PMC8444284; doi:10.15252/msb.202010105)
Supplement: Supplementary file 5 — Source Data for Figure 3 [file MSB-17-e10105-s001.zip › Figure3A_sourcedata/GSEA_3065/nosigdb_state4.GseaPreranked.1622718430994/LARSSON_STATE_3.html]

Details for gene set LARSSON\_STATE\_3[GSEA]

|  || Dataset | state4 |
| Phenotype | NoPhenotypeAvailable |
| Upregulated in class | na\_neg |
| GeneSet | LARSSON\_STATE\_3 |
| Enrichment Score (ES) | -0.831665 |
| Normalized Enrichment Score (NES) | -2.945513 |
| Nominal p-value | 0.0 |
| FDR q-value | 0.0 |
| FWER p-Value | 0.0 |
Table: GSEA Results Summary

  

Fig 1: Enrichment plot: LARSSON\_STATE\_3      
 Profile of the Running ES Score & Positions of GeneSet Members on the Rank Ordered List

  

| PROBE | GENE SYMBOL | GENE\_TITLE | RANK IN GENE LIST | RANK METRIC SCORE | RUNNING ES | CORE ENRICHMENT || 1 | LPL |  |  | 3121 | 0.023 | -0.5042 | No |
| 2 | GPNMB |  |  | 3153 | 0.022 | -0.5083 | No |
| 3 | SPTBN1 |  |  | 3155 | 0.022 | -0.5075 | No |
| 4 | SLC44A1 |  |  | 3731 | 0.004 | -0.6004 | No |
| 5 | ALCAM |  |  | 4499 | -0.045 | -0.7227 | No |
| 6 | MYH10 |  |  | 4880 | -0.074 | -0.7811 | No |
| 7 | SCRG1 |  |  | 4970 | -0.080 | -0.7921 | No |
| 8 | ITM2B |  |  | 5050 | -0.087 | -0.8012 | No |
| 9 | APP |  |  | 5192 | -0.101 | -0.8198 | No |
| 10 | FGFBP3 |  |  | 5233 | -0.104 | -0.8218 | No |
| 11 | DST |  |  | 5242 | -0.105 | -0.8187 | No |
| 12 | PON2 |  |  | 5246 | -0.105 | -0.8147 | No |
| 13 | ABHD2 |  |  | 5255 | -0.107 | -0.8115 | No |
| 14 | PTN |  |  | 5261 | -0.107 | -0.8078 | No |
| 15 | GPC1 |  |  | 5348 | -0.113 | -0.8169 | No |
| 16 | NCAM1 |  |  | 5391 | -0.117 | -0.8187 | No |
| 17 | IQGAP1 |  |  | 5446 | -0.124 | -0.8222 | No |
| 18 | ATP6AP2 |  |  | 5453 | -0.125 | -0.8179 | No |
| 19 | KCNQ1OT1 |  |  | 5539 | -0.135 | -0.8259 | Yes |
| 20 | GPM6A |  |  | 5563 | -0.137 | -0.8239 | Yes |
| 21 | WLS |  |  | 5572 | -0.139 | -0.8193 | Yes |
| 22 | SOX6 |  |  | 5612 | -0.146 | -0.8194 | Yes |
| 23 | TM9SF3 |  |  | 5651 | -0.152 | -0.8191 | Yes |
| 24 | DYNC1H1 |  |  | 5710 | -0.162 | -0.8217 | Yes |
| 25 | MMP16 |  |  | 5734 | -0.168 | -0.8183 | Yes |
| 26 | CANX |  |  | 5755 | -0.173 | -0.8142 | Yes |
| 27 | RPN2 |  |  | 5771 | -0.178 | -0.8091 | Yes |
| 28 | MALAT1 |  |  | 5783 | -0.179 | -0.8033 | Yes |
| 29 | RTN3 |  |  | 5793 | -0.181 | -0.7971 | Yes |
| 30 | LAMC1 |  |  | 5795 | -0.182 | -0.7895 | Yes |
| 31 | PTPRZ1 |  |  | 5811 | -0.186 | -0.7841 | Yes |
| 32 | B2M |  |  | 5823 | -0.188 | -0.7779 | Yes |
| 33 | TMBIM6 |  |  | 5855 | -0.196 | -0.7746 | Yes |
| 34 | FAT1 |  |  | 5898 | -0.206 | -0.7727 | Yes |
| 35 | HMGCR |  |  | 5906 | -0.208 | -0.7650 | Yes |
| 36 | HACD3 |  |  | 5916 | -0.211 | -0.7576 | Yes |
| 37 | PLD5 |  |  | 5924 | -0.213 | -0.7497 | Yes |
| 38 | SSR4 |  |  | 5941 | -0.218 | -0.7430 | Yes |
| 39 | SPCS3 |  |  | 5948 | -0.220 | -0.7347 | Yes |
| 40 | CCDC47 |  |  | 5970 | -0.227 | -0.7284 | Yes |
| 41 | LAPTM4B |  |  | 5994 | -0.236 | -0.7222 | Yes |
| 42 | MDM4 |  |  | 6003 | -0.239 | -0.7133 | Yes |
| 43 | HNRNPU |  |  | 6008 | -0.241 | -0.7038 | Yes |
| 44 | EMC7 |  |  | 6020 | -0.246 | -0.6952 | Yes |
| 45 | CALU |  |  | 6049 | -0.268 | -0.6883 | Yes |
| 46 | BSG |  |  | 6050 | -0.269 | -0.6770 | Yes |
| 47 | PGRMC1 |  |  | 6051 | -0.270 | -0.6655 | Yes |
| 48 | HSPA5 |  |  | 6061 | -0.274 | -0.6554 | Yes |
| 49 | HSP90AA1 |  |  | 6062 | -0.275 | -0.6437 | Yes |
| 50 | GRIA2 |  |  | 6065 | -0.277 | -0.6323 | Yes |
| 51 | VGF |  |  | 6066 | -0.277 | -0.6206 | Yes |
| 52 | PLAT |  |  | 6079 | -0.285 | -0.6105 | Yes |
| 53 | EMP3 |  |  | 6085 | -0.287 | -0.5991 | Yes |
| 54 | SYNE2 |  |  | 6086 | -0.287 | -0.5869 | Yes |
| 55 | APLP2 |  |  | 6087 | -0.288 | -0.5747 | Yes |
| 56 | CD320 |  |  | 6088 | -0.289 | -0.5625 | Yes |
| 57 | LRPAP1 |  |  | 6096 | -0.294 | -0.5512 | Yes |
| 58 | STT3B |  |  | 6101 | -0.298 | -0.5392 | Yes |
| 59 | TMED9 |  |  | 6102 | -0.298 | -0.5266 | Yes |
| 60 | PIF1 |  |  | 6106 | -0.301 | -0.5143 | Yes |
| 61 | HSP90AB1 |  |  | 6119 | -0.312 | -0.5030 | Yes |
| 62 | DSEL |  |  | 6121 | -0.316 | -0.4899 | Yes |
| 63 | SQLE |  |  | 6123 | -0.316 | -0.4766 | Yes |
| 64 | PDIA3 |  |  | 6124 | -0.317 | -0.4632 | Yes |
| 65 | LMAN1 |  |  | 6130 | -0.322 | -0.4504 | Yes |
| 66 | PRKDC |  |  | 6149 | -0.341 | -0.4388 | Yes |
| 67 | CALR |  |  | 6153 | -0.344 | -0.4248 | Yes |
| 68 | PRDX4 |  |  | 6155 | -0.345 | -0.4104 | Yes |
| 69 | FUS |  |  | 6170 | -0.371 | -0.3969 | Yes |
| 70 | CD9 |  |  | 6176 | -0.378 | -0.3817 | Yes |
| 71 | MANF |  |  | 6186 | -0.400 | -0.3662 | Yes |
| 72 | NASP |  |  | 6187 | -0.403 | -0.3492 | Yes |
| 73 | TIMP1 |  |  | 6191 | -0.409 | -0.3323 | Yes |
| 74 | SPCS2 |  |  | 6193 | -0.419 | -0.3148 | Yes |
| 75 | MTRNR2L12 |  |  | 6202 | -0.451 | -0.2970 | Yes |
| 76 | BCAN |  |  | 6211 | -0.481 | -0.2779 | Yes |
| 77 | PDIA6 |  |  | 6213 | -0.482 | -0.2576 | Yes |
| 78 | CD63 |  |  | 6220 | -0.496 | -0.2376 | Yes |
| 79 | HSP90B1 |  |  | 6225 | -0.509 | -0.2167 | Yes |
| 80 | TFPI2 |  |  | 6230 | -0.518 | -0.1954 | Yes |
| 81 | GGH |  |  | 6232 | -0.522 | -0.1735 | Yes |
| 82 | ASPM |  |  | 6254 | -0.680 | -0.1481 | Yes |
| 83 | POSTN |  |  | 6256 | -0.753 | -0.1164 | Yes |
| 84 | TOP2A |  |  | 6260 | -0.814 | -0.0824 | Yes |
| 85 | MKI67 |  |  | 6261 | -0.882 | -0.0451 | Yes |
| 86 | CENPF |  |  | 6263 | -1.068 | 0.0000 | Yes |
Table: GSEA details [plain text format]

  

Fig 2: LARSSON\_STATE\_3: Random ES distribution      
 Gene set null distribution of ES for **LARSSON\_STATE\_3**
